# Melatonin in preservation solutions prevents ischemic injury in rat kidneys

**DOI:** 10.1371/journal.pone.0273921

**Published:** 2022-08-31

**Authors:** Abdurrahman Coskun, Cumhur Yegen, Serap Arbak, Wafi Attaallah, Omer Gunal, Merve Acikel Elmas, Yasemin Ucal, Ozge Can, Banu Baş, Zeynep Yildirim, Ismail Seckin, Sibel Demirci, Mustafa Serteser, Aysel Ozpinar, Ahmet Belce, Gulcin Basdemir, Derya Emel Moldur, Ecenur Izzete Derelioglu, Tahir Koray Yozgatli, Yigit Erdemgil, Ibrahim Unsal

**Affiliations:** 1 Department of Medical Biochemistry, Acibadem Mehmet Ali Aydinlar University, School of Medicine, Istanbul, Turkey; 2 Acibadem Labmed Clinical Laboratories, Istanbul, Turkey; 3 Department of General Surgery, Marmara University, School of Medicine, Istanbul, Turkey; 4 Department of Histology and Embryology, Acibadem Mehmet Ali Aydinlar University, School of Medicine, Istanbul, Turkey; 5 Faculty of Engineering, Department of Medical Engineering, Acibadem Mehmet Ali Aydinlar University, Istanbul, Turkey; 6 Department of Histology and Embryology, Istanbul University Cerrahpasa, Cerrahpasa School of Medicine, Istanbul, Turkey; 7 Department of Histology and Embryology, Biruni University, School of Medicine, Istanbul, Turkey; 8 Department of Medical Biochemistry, Biruni University, School of Medicine, Istanbul, Turkey; 9 Department of Pathology, Memorial Hospital, Istanbul, Turkey; 10 Acibadem Mehmet Ali Aydinlar University, School of Medicine, Istanbul, Turkey; University of Bari: Universita degli Studi di Bari Aldo Moro, ITALY

## Abstract

Transplantation is lifesaving and the most effective treatment for end-stage organ failure. The transplantation success depends on the functional preservation of organs prior to transplantation. Currently, the University of Wisconsin (UW) and histidine-tryptophan-ketoglutarate (HTK) are the most commonly used preservation solutions. Despite intensive efforts, the functional preservation of solid organs prior to transplantation is limited to hours. In this study, we modified the UW solution containing components from both the UW and HTK solutions and analyzed their tissue-protective effect against ischemic injury. The composition of the UW solution was changed by reducing hydroxyethyl starch concentration and adding Histidine/Histidine-HCl which is the main component of HTK solution. Additionally, the preservation solutions were supplemented with melatonin and glucosamine. The protective effects of the preservation solutions were assessed by biochemical and microscopical analysis at 2, 10, 24, and 72 h after preserving the rat kidneys with static cold storage. Lactate dehydrogenase (LDH) activity in preservation solutions was measured at 2, 10, 24, and 72. It was not detectable at 2 h of preservation in all groups and 10 h of preservation in modified UW+melatonin (mUW-m) and modified UW+glucosamine (mUW-g) groups. At the 72^nd^ hour, the lowest LDH activity (0.91 IU/g (0.63–1.17)) was measured in the mUW-m group. In comparison to the UW group, histopathological damage score was low in modified UW (mUW), mUW-m, and mUW-g groups at 10, 24, and 72 hours. The mUW-m solution at low temperature was an effective and suitable solution to protect renal tissue for up to 72 h.

## Introduction

Organ transplantation is a powerful therapeutic approach for end-stage organ failure [1] and there is an extreme necessity for high-quality organ supply [2]. Globally, the shortage of organs is a major public health problem and according to World Health Organization (WHO), less than 10% of organ demands are being met [3,4].

The success of transplantation does not only depend on surgical techniques, postoperative care, and effective immunosuppressive agents but also on the functional preservation of organs prior to transplantation.

After cessation of circulation, the absence of blood flow causes complete ischaemic injury in solid organs [5]. In comparison to pure hypoxic injury, complete ischemia causes more severe and rapid intracellular acidosis [6] due to the lack of oxygen and several essential metabolic substrates, and the accumulation of waste products resulting in progressive damage that is limiting the life span of organs to a very short period (i.e., 30–60 min) [1]. The lack of oxygen induces anaerobic metabolism, leads to dysfunction of the mitochondrial oxidative phosphorylation, and decreases ATP synthesis which causes the failure of sodium-potassium (Na^+^-K^+^-ATPase) [7] and calcium pumps (Ca^2+^-ATPase) [8]. The inhibition of sodium-potassium pumps destroys the intracellular and extracellular ionic balance which causes the retention of sodium within cells and potassium out of cells. Moreover, an increased concentration of intracellular sodium decreases the activity of sodium–hydrogen pumps (Na^+^-H^+^ pumps) [9]. Due to the failure of pumps, the intracellular sodium, hydrogen, and calcium levels are elevated which causes hyperosmolarity and leads to water flow into the cell and cell swelling. The retention of hydrogen ions and increased intracellular acidosis lead to impaired enzyme activity and detachment of ribosomes [10] which over time induces cell deaths. The detailed information regarding ischemia and reperfusion injury of transplantation process can be found in [11].

To protect organs efficiently, hypothermia is the first line preservation method employed prior to transplantation. In human metabolism, cooling organs by 10 °C decreases the biochemical reaction rate by 1.5–2.0 [1]. For biological systems, the relationship between temperature and reaction velocities can be expressed by the van’t Hoff coefficient Q_10_ [12] as given below:

$$Q_{10}={\left(\frac{K_{2}}{K_{1}}\right)}^{\left(\frac{10}{T_{2}-T_{1}}\right)}$$

where k_1_ and k_2_ are the reaction rates at T_1_ and T_2_ degrees respectively. Using van’t Hoff coefficient Q_10_, it can be shown that cooling organ to 0 °C slowed biochemical reaction velocities by a factor of 12.

Static cold storage (SCS) is the widely applied preservation method of solid organs. In SCS, the organs are flushed with the preservation solution at low temperatures (i.e., 2ºC-4ºC) and retained in a cooled preservation solution until transplantation. Although hypothermia suppresses the metabolic rate of organs and reduces oxygen and nutrient requirements however, when used alone, it does not protect solid organs adequately for long period. Furthermore, prolonged hypothermia has deleterious effects on cellular metabolism which promotes delayed graft function (DGF) [13]. Thus, for functional preservation, pharmacological intervention i.e. preservation solutions is essential.

The preservation solutions are aimed at protecting the biochemical and physiological conditions of the pre-transplanted organs and thus, minimizing the catastrophic effects of a hypoxic and cold environment. Preservation solutions mainly contain 1) osmotically active substances to prevent cell swelling, 2) electrolytes to stabilize cell membranes and maintain osmotic gradient between extracellular and intracellular spaces, 3) buffers to maintain pH gradient, 4) colloids to prevent interstitial edema, 5) antioxidants to prevent reperfusion injury and 6) nutrients for supporting cellular energy [1].

The innovations for preservation solutions were initiated by Collins et al. in 1969 [14]. Since then several molecules such as; sucrose, mannitol, free radical scavengers, buffers, and others have been tested in different preservation solutions [15–17]. However, only a few preservation solutions are used in clinical settings. Currently, the University of Wisconsin (UW) and histidine-tryptophan-ketoglutarate (HTK) are the most commonly used preservation solutions [18].

In UW solution the osmotic concentration is sustained by metabolically inactive substrates and hydroxyethyl starch (HES) as colloid carrier [18]. The colloidal structures included in the preservation solution resulted in an adequate response to the extravasation of fluids caused by hydrostatic pressure. Therefore, the UW solution is widely used in many centers for multi-organ transplantations. Although the UW solution has been accepted as the ‘gold standard’ in solid organ preservation, the main disadvantage of the solution is its high viscosity [18].

At the beginning of the 1980s, HTK was entered into the organ transplantation field as an alternative preservation solution. It has a very low viscosity which increases its perfusion to tissue and contains histidine which is a potent buffer system [17,19].

Despite the great success of UW and HTK in graft survival and acute rejection [20], both solutions have limitations in different aspects. Consequently, the functional preservation period is not at the desired level and is still limited to hours (h). Therefore, we hypothesized that a new solution containing components from both the UW and HTK solution, supplemented with powerful antioxidant and tissue protective agents, with a lower viscosity and a higher buffering effect than the UW solution, could better protect the kidneys against ischemic injury.

Melatonin (N-acetyl-5-methoxytryptamine), a powerful candidate that can be used in this field [21], is synthesized mainly by the pineal gland and has anti-inflammatory, anti-oxidative, anti-apoptotic effects and has crucial functions in immune and endocrine systems, circadian rhythms, and sleep regulations [22–24].

Glucosamine (GlcN), an amino sugar synthesized in the human body, has crucial structural and metabolic functions. It is the essential component of various macromolecules such as glycoproteins and glycosaminoglycans which are essential for maintaining tissue integrity. Additionally, it has anti-oxidant, anti-fibrotic, anti-inflammatory, and cardioprotective effects [25].

In our previous studies we showed that, during preservation prior to transplantation, various structural peptides and proteins are released from tissue to the preservation solution [26,27]. These findings suggested that the destruction is not limited to cellular components but also to extracellular infrastructures that maintain the integrity of the tissue. We thought that GlcN might prevent the destruction of structural components as it is an important component of glycolipids, glycoproteins, and proteoglycans which have various cellular and extracellular functions [28].

In the present study, we changed the composition of the UW solution by 1) reducing the amount of HES and adding histidine due to its well-known efficacy in HTK solution and 2) adding melatonin and GlcN as powerful protective agents. We analyzed the protective effect of these alterations on rat kidneys preserved in different preservation solutions using biochemical, proteomic, and microscopic techniques.

## Materials and methods

### Materials

All chemicals and reagents were purchased from Sigma Aldrich (St Louis, MO, USA) unless otherwise stated. The two-dimensional (2D) electrophoresis equipment, i.e., isoelectric focusing (PROTEAN IEF) and sodium dodecyl sulfate-polyacrylamide gel electrophoresis (SDS-PAGE) (Criterion Dodeca Cell, 12 gels), silver stain kit, dithiothreitol (DTT), iodoacetamide (IAA), ReadyStrip IPG strips pH 3–10 nonlinear 11 cm, 8–16% Criterion Tris-HCl Gel, glycine, SDS, tris, urea, and Bio-lyte 3/10 ampholytes were obtained from Bio-Rad (Hercules, CA, USA). Tetramethylethylenediamine, ethanol, and methanol were from Merck (Whitehouse Station, NJ, USA).

### Preparation of preservation solution

In this study, we used six different preservation solutions. The dosage of melatonin [29] and glucosamine [30] and the details of the solutions are given in Table 1. Since melatonin is a light-sensitive substance, it was added to the preservation solutions just before the experimental procedure and stored in a dark place.

**Table 1:** Preservation solutions used in experimental studies.

Groups	Preservation solutions
**Group 1**	University of Wisconsin Solution (UW)
**Group 2**	UW + Melatonin (30 mg/L)
**Group 3**	UW + Glucosamine (20 mg/L)
**Group 4**	Modified UW*
**Group 5**	Modified UW* + Melatonin (30 mg/L)
**Group 6**	Modified UW*+ Glucosamine (20 mg/L)

*: The composition of modified UW is given in Table 2.

We modified the UW solution in our laboratory, by decreasing the concentration of HES by 20% and adding Histidine/Histidine-HCl. The final concentrations of chemical components in the modified UW solution (mUW) are given in Table 2.

**Table 2:** The composition of modified UW solution used in experimental studies.

Components	Standard UW Solution	Modified UW Solution
**Raffinose**	30 mmol/L	30 mmol/L
**K-Lactobionate**	100 mmol/L	100 mmol/L
H_2_PO_4_^-^	5 mmol/L	5 mmol/L
**HPO42-**	20 mmol/L	20 mmol/L
**HES (Hydroxyethyl starch)** *	5% g	4% g
**SO42-**	5 mmol/L	5 mmol/L
**Adenosine**	5 mmol/L	5 mmol/L
**Glutathione**	3 mmol/L	3 mmol/L
**Allopurinol**	1 mmol/L	1 mmol/L
**Na** ^+^	30 mmol/L	30 mmol/L
**K** ^+^	120 mmol/L	120 mmol/L
**Mg** ^+^	5 mmol/L	5 mmol/L
**Histidine/Histidine- HCl** **	NA	30/3 mmol/L
**pH**	7.4	7.4
**Osmolarity**	320 mOsm	358 mOsm

*: The concentration of HES decreased by %20;

**: Histidine/Histidine- HCl was added to standard UW solution.

### Animals

A total of 130 Wistar albino male rats weighing 350–400 gr were used. All animals were housed with a 12-h light/12-h dark cycle at 22°C and 55%±5% relative humidity. Water and food were provided *ad libitum*. All animal experiments were conducted at Acibadem Mehmet Ali Aydinlar University. The study was approved by the ethical committee of Acibadem Mehmet Ali Aydinlar University (ACU HADYEK 2014/5) and carried out in compliance with the ARRIVE guidelines. All procedures were performed in accordance with relevant guidelines and regulations.

### Surgical procedures

We used a standard surgical procedure throughout the study. Briefly, the rats were anesthetized with intraperitoneal 50 mg/kg ketamine (Ketalar, Eczacıbaşı, Istanbul/Turkey). After midline laparotomy, the abdominal aorta and infrarenal abdominal aorta were suspended using 2/0 silk sutures. A 23-gauge polyethylene tube (cannula) was placed within the infrarenal abdominal aorta and 0.5 mL blood samples were taken from the cannula. To prevent dehydration, ringer lactate was infused via the abdominal aorta with slow and constant velocity. Following the 2/0 silk suture which was placed at the level of the hiatus was ligated to the upper abdominal aorta and blood circulation from the heart to the kidneys was discontinued. After the lateral incision to the infra-renal vena cava inferior, blood and perfusion solution was allowed to exsanguinate. The perfusion was continued for 1–2 minutes until the vena cava received non-blood ringer lactate and both kidneys were completely paled. The kidneys were then perfused using a preservation solution through the aortic cannula. When the perfusion was complete, a bilateral nephrectomy was performed and both kidneys were removed gently.

The kidneys were purged from perirenal adipose tissue and blood clots adhering to the capsule. After weighing them, the tissues were immediately placed in the sterile container with 20 mL preservation solutions and kept at +4ºC until analysis.

Throughout the entire surgical operation, the left kidneys were removed before the right ones. The ischemia times of the right kidneys were approximately 2 min longer than the left kidneys. Surgical procedures were performed by two experienced surgeons. The task distribution among researchers was performed in all other procedures. Throughout the entire study, everyone maintained the same task to ensure standardization.

### Biochemical analysis

Two mL of preservation solutions from each group were collected at 2, 10, 24, and 72 h of preservation time. The lactate dehydrogenase (LDH) activity and total protein levels were measured from all preservation solutions by standard clinical chemistry methods using Siemens Advia 1800. LDH activity and protein concentration were expressed as IU/g and mg/g wet tissue respectively. All samples were analyzed within one run to prevent possible bias. The osmolarity of the modified solution was measured using freezing point depression method (Osmometer, Advanced Instruments, MA, USA).

#### Proteomics analysis

Two ml samples were collected from each organ preservation solution at four different time points (2, 10, 24, and 72 h). The samples were centrifuged at 5000x*g* and supernatant was stored at -80 ºC until analyses. The two-dimensional gel electrophoresis protocol was adapted from Adams and Gallagher [31]. The deviations from the protocol were at the isoelectric focusing using the following protocol: 200 V for 15 min, 1000 V (gradient) for 3 h, 10000 V for 3 h, for a total of 55000 V, and separation in the second dimension performed at 150 V for 7 h. The silver-stained gels were scanned by the ChemiDoc MP imaging system (BioRad, Hercules, CA, USA). The samples obtained at the latest preservation time point (72 h) for each solution, were mixed to obtain master gels.

The common protein spots among different preservation solutions were excised manually. The protein identifications and peptide detections are based on the protocol by Webster and Oxley [32] using Autoflex MALDI-TOF/TOF instrument (Bruker Daltonics) in positive reflectron mode. The acquisition parameters were: laser 70%, frequency: 60, mass range acquisition: 500–3500 Da, number of shots: 500, sample rate: 0.5 GS/S, electronic gain: 100 Mv. The Mascot database (Matrix Science, London, UK) was used to search the SwissProt 2013_02 database with the following parameters: taxonomy: Rodents, enzyme: trypsin, global modifications: carbamido methyl cysteine, mass values: MH+ monoisotopic, maximum missed cleavage sites: 1, peptide charge: 1 H+, mass tolerance: 100 ppm.

### Histopathological characterization

#### Light microscope analysis

Kidney tissue samples (5 samples from each group) were fixed in a 10% neutral buffered formalin solution. Following the fixation, dehydration, and clearing, tissues were embedded in paraffin. Sections of approximately 5 μm of thickness were stained with Haematoxylin–Eosin (H&E) and Periodic acid-Schiff (PAS) reaction. Renal injury, based on glomerular and tubular degeneration, vasocongestion, and inflammatory cell infiltration was scored light microscopically by using a scale ranging from 0 to 3 (0: none, 1: mild, 2: moderate, and 3: severe) for each criterion [33]. The total score was 12. For each slide, 20 similar regions were evaluated by 2 histologists who were blinded to the experimental groups.

#### Electron microscope analysis

Kidney tissue samples were fixed with 2.5% glutaraldehyde in PBS (0.1 M, pH 7.2), then post-fixed in 1% osmium tetroxide in PBS (0.1 M, pH 7.2), dehydrated, and cleared with an automated tissue processor (Leica EM TP). The samples were then embedded in Epon 812 resin (Fluka, Sigma–Aldrich Chemica, Steinheim, Switzerland). Approximately 1 μm of semi-thin sections were stained with toluidin blue (TB). Ultrathin sections (Approximately 60 nm) were stained with uranyl acetate and lead citrate. Ultrathin sections were investigated using a Jeol JEM 1011 transmission electron microscope (TEM) (Tokyo, Japan) and photographed. For TEM evaluation, 10 similar glomerular basement membrane (GBM) and proximal tubule basal lamina (PTBL) regions from kidney specimens of 72^nd^ h of the experimental groups were evaluated based on GBM and PTBL. The thicknesses of GBM and PTBL were measured from randomly selected 10 grids belonging to different rats.

### Statistical analysis

The number of animals included in the study was calculated using the method proposed by Arifin et al [34] and Serdar et al [35]. The normality of data was evaluated by the Anderson-Darling test. Kruskal-Wallis test was used to assess the differences among groups. If the groups significantly differed, the Mann Whitney U test was further used for each pair to find the significantly different group(s). *P*<0.05 was considered statistically significant.

## Results

### Biochemical measurements

#### Lactate dehydrogenase activity in preservation solution

Lactate dehydrogenase (LDH) activity was measured in six different preservation solutions at 2, 10, 24, and 72 h of preservation time. As shown in Table 3, LDH activity was not detected at 2 h of preservation in all groups. However, at 10 h of preservation, it was measurable in all groups but not in the mUW-m and mUW-g groups. At 24 h, although statistically not significant, the LDH activity of all groups was lower than the LDH activity of the UW group. At 72 h the lowest median (interquartile range (IQR)) activity was detected in the mUW-m (0.91 IU/g (0.63–1.17)) and the highest median (IQR) activity was detected in the mUW-g group (2.29 IU/g (1.27–2.48)).

**Table 3:** LDH activity (IU/g wet tissue) was measured from six different preservation solutions at 2, 10, 24, and 72 h of preservation time. Data are given as a median and interquartile range (IQR).

Preservation Solutions, n = 10	Preservation Time			
	2. h	10. h	24. h	72. h
**UW**	ND	0.27 (0.22–0.60)	0.52 (0.29–0.59)	1.11 (0.89–1.69)*
**UW + Melatonin**	ND	0.31 (0.30–0.39)	0.39 (0.24–0.71)	1.24 (0.62–1.71)
**UW + Glucosamine**	ND	0.25 (0.23–0.29)	0.41 (0.30–0.57)	1.53 (1.10–2.45)
**Modified UW**	ND	0.34 (0.22–0.44)	0.40 (0.29–0.54)	2.00 (1.39–2.49)^a^^,^^b^^,^^d^
**Modified UW + Melatonin**	ND*	ND	0.40 (0.30–0.50)	0.91 (0.63–1.17)^c^^,^^e^^,^*
**Modified UW + Glucosamine**	ND	ND	0.35 (0.25–0.44)	2.29 (1.27–2.48)^f^^,^^g^

g: Gram; ND: Not detected;

^a^: Different from UW at 72. Hour (p = 0.006);

^b^: Different from UW-m at 72 hour (p = 0.015);

^c^: Different from UW-g at 72 hour (p = 0.004);

^d^: Different from mUW-m at 72 hour (p = 0.0001);

^e^: Different from mUW-g at 72 hour(p = 0.0003);

^f^: Different from UW at 72. Hour (p = 0.006);

^g^: Different from UW-m at 72 hour (p = 0.015);

*:n = 11.

### Total protein in preservation solution

The total protein levels measured from six different preservation solutions at 2, 10, 24, and 72 h of preservation time are given in Table 4. At 2 h, although statistically not significant, the lowest median (IQR) protein concentration was measured in mUW-m group (0.20 mg/g (0.15–0.25)). Similarly, at 10 h, the lowest median (IQR) protein concentration was measured in the mUW-m group (0.25 mg/g (0.20–0.27)) and this was significantly lower than the mUW-g group (0.47 mg/g (0.30–0.53)). At 24 h, the lowest median (IQR) protein concentration was measured in the UW-g group (0.29 mg/g (0.24–0.39)) and this was significantly lower than the mUW and mUW-g group. The mUW and mUW-g group protein levels were higher than the other groups (Table 4). However, the addition of melatonin to the mUW group significantly decreased the total protein levels (median (IQR): 0.31 mg/g (0.27–0.39)).

**Table 4:** Protein concentration (mg/g wet tissue) was measured from six different preservation solutions at 2, 10, 24, and 72 h of preservation time. Data are given as median and interquartile range (IQR).

Preservation Solutions, n = 10	Preservation Time			
	2. h	10. h	24. h	72. h
**UW**	0.23 (0.16–0.30)	0.29 (0.26–0.64)	0.38 (0.29–0.54)	0.62 (0.46–0.70)*
**UW + Melatonin**	0.32 (0.26–0.43)	0.29 (0.20–0.37)	0.32 (0.29–0.34)	0.67 (0.48–0.89)
**UW + Glucosamine**	0.24 (0.15–0.28)	0.32 (0.24–0.39)	0.29 (0.24–0.39)	0.45 (0.39–0.62)
**Modified UW**	0.23 (0.17–0.34)	0.31 (0.25–0.39)	0.46 (0.33–0.64)^c^^,^^d^^,^^e^	0.40 (0.26–0.81)
**Modified UW + Melatonin**	0.20 (0.15–0.25)*	0,25 (0.20–0.27)	0.31 (0.27–0.39)	0.40 (0.34–0.52)^j^^,^*
**Modified UW + Glucosamine**	0.28 (0.25–0.31)	0.47 (0.30–0.53)^a^^,^^b^	0.73 (0.40–3.38)^f^^,^^g^^,^^h^	0.81 (0.53–0.99)^i^^,^^k^^,^^l^^,^^m^

g: Gram;

^a^: Different from UW-m at 10 hour (p = 0.035);

^b^: Different from mUW-m at 10 hour (p = 0.002);

^c^: Different from UW-g at 24 hour (p = 0.044);

^d^: Different from UW-m at 24 hour (p = 0.015);

^e^: Different from mUW-m at 24 hour (p = 0.036);

^f^: Different from UW-g at 24 hour (p = 0.002);

^g^: Different from UW-m at 24 hour (p = 0.0001);

^h^: Different from mUW-m at 24 hour (p = 0.001);

^i^: Different from UW at 72 hour (p = 0.043);

^j^: Different from UW-m at 72 hour (p = 0.043);

^k^: Different from UW-g at 72 hour (p = 0.003);

^l^: Different from mUW at 72 hour (p = 0.043);

^m^: Different from mUW-m at 72 hour (p = 0.0015);

*:n = 11.

At 72 h the lowest protein level was measured in the mUW-m group (median (IQR): 0.40 mg/g (0.34–0.52)). This was significantly lower than UW-m and mUW-g groups.

#### Protein profiles released from kidney tissues to preservation solution

We used a gel-based proteomics approach to analyze the type of proteins and peptides released from tissue to the preservation medium. Among the common protein spots detected in different preservation solutions, 29 different proteins were identified (Table 5).

**Table 5:** Proteins belonging to rat kidney tissues detected from preservation solutions.

Proteins and Peptides	Entry Name (UniProt)	Gene Name	Accession Number (UniProt)
Alpha-1-antiproteinase	A1AT_RAT	Serpina1	P17475
Major urinary protein	MUP_RAT	N/A	P02761
Mitochondrial import inner membrane translocase subunit Tim8 A	TIM8A_RAT	Timm8a	Q9WVA1
Beta-crystallin B2	CRBB2_RAT	Crybb2	P62697
Peroxiredoxin-6	PRDX6_RAT	Prdx6	O35244
Ethanolamine-phosphate cytidylyltransferase	PCY2_RAT	Pcyt2	O88637
FAS-associated factor 2	FAF2_RAT	Faf2	Q5BK32
Serum albumin	ALBU_RAT	Alb	P02770
Aspartate-tRNA ligase, cytoplasmic	SYDC_RAT	Dars	P15178
Aminoacylase-1A	ACY1A_RAT	Acy1a	Q6AYS7
Fascin	FSCN1_RAT	Fscn1	P85845
Transmembrane protein 43	TMM43_RAT	Tmem43	Q5XIP9
Fidgeting-like protein 1	FIGL1_RAT	Fignl1	Q6GX84
Serotransferrin	TRFE_RAT	Tf	P12346
Transketolase	TKT_RAT	Tkt	P50137
Adenosine monophosphate-protein transferase FICD	FICD_RAT	Ficd	Q6AY47
Argininosuccinate synthase	ASSY_RAT	Ass1	P09034
Alpha-ketoglutarate-dependent dioxygenase alkB homolog 3	ALKB3_RAT	Alkbh3	Q5XIC8
Alcohol dehydrogenase [NADP(+)]	AK1A1_RAT	Akr1a1	P51635
Porphobilinogen deaminase	HEM3_RAT	Hmbs	P19356
Isoaspartyl peptidase/L-asparaginase	ASGL1_RAT	Asrgl1	Q8VI04
Protein THEM6	THEM6_RAT	Them6	Q5XIE1
Ras-related protein Rab-1B	RAB1B_RAT	Rab1b	P10536
Midkine	MK_RAT	Mdk	Q9R1S9
C-type lectin domain family 2 member D3	CL2D3_RAT	Clec2d3	A4KWA6
Cyclin-A1	CCNA1_RAT	Ccna1	Q6AY13
Ras-related protein Rab-19	RAB19_RAT	Rab19	Q5M7U5

#### Osmolarity of the Modified UW solution

The osmolarity of the modUW solution was measured as 358 mOsm and was higher than the osmolatity of the standard UW solution (320 mOsm).

### Microscopic examinations

#### Light microscopical investigations

**Fig 1:**
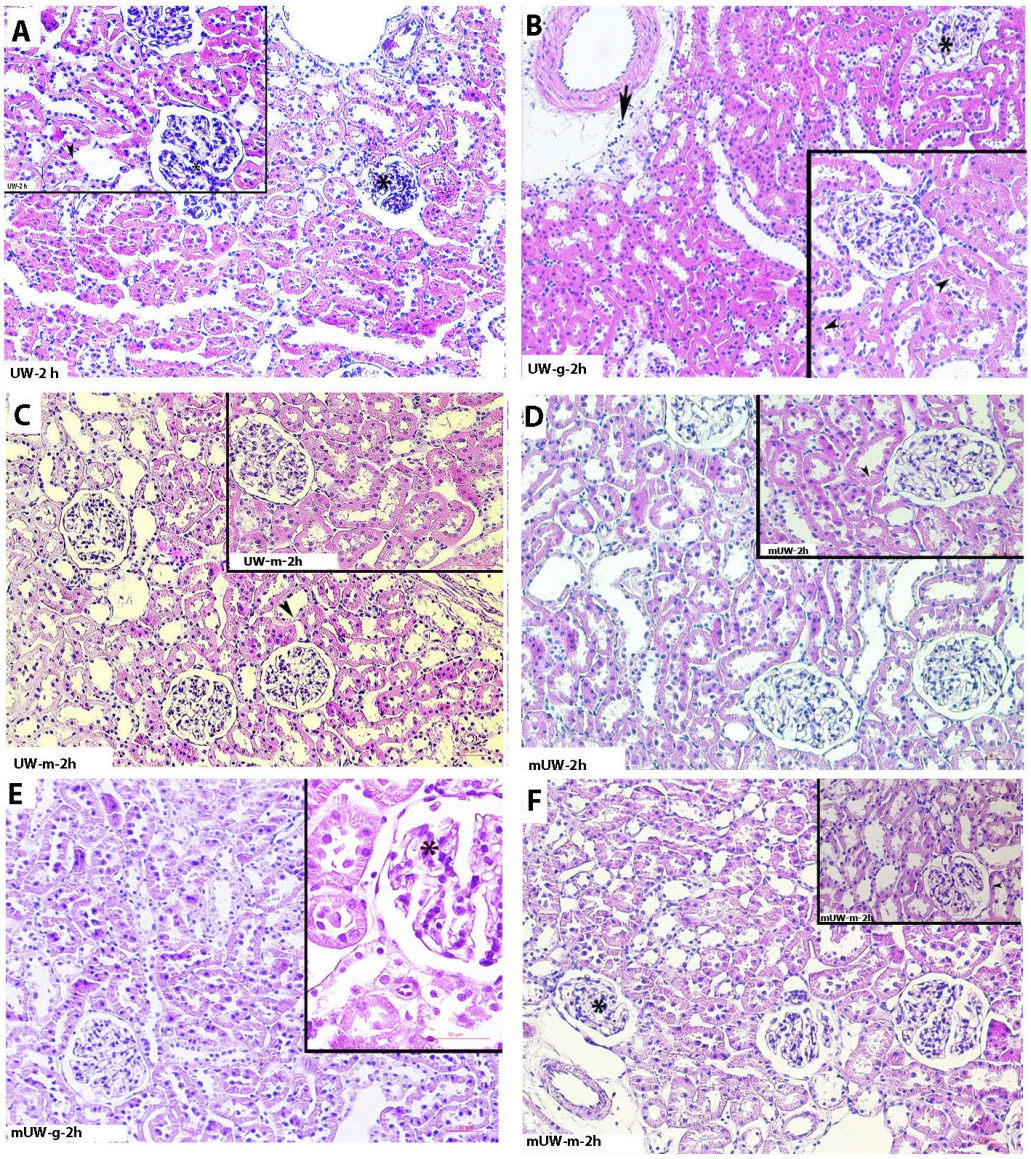
Photomicrographs of kidney tissues stored for 2 hours in experimental kidney preservation solutions. Slight renal tissue degeneration at the 2nd hour of the experimental groups. Glomerular (asterisk) and tubular (arrowhead) degenerations in UW **(A**), UW-g **(B)**, UWm **(C)**, mUW **(D)**, mUW-g **(E)** and mUW-m **(F)** groups. H&E staining, A,B,C,D,E,F x200 magnification, insets: x400 magnification.

**Fig 2:**
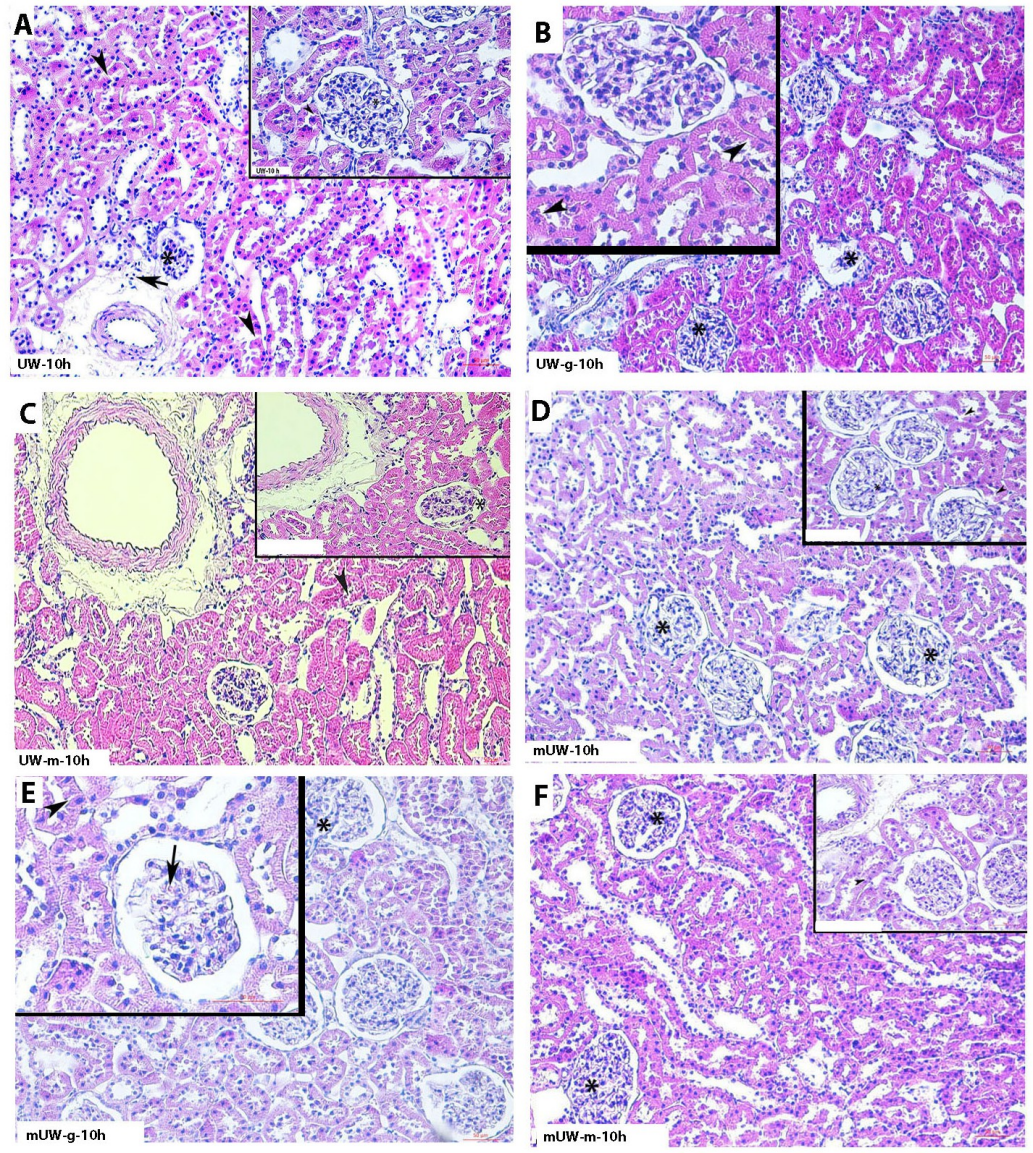
Photomicrographs of kidney tissues stored for 10 hours in experimental kidney preservation solutions. Relatively high degree of glomerular (asterisk) and tubular degeneration (arrowhead) were seen in UW **(A)** UW-g **(B)**, UW-m**(C)**, mUW**(D)** mUW-g **(E)** and mUW-m **(F)** groups. H&E staining, A,B,C,D,E,F x200 magnification, insets: x400 magnification.

**Fig 3:**
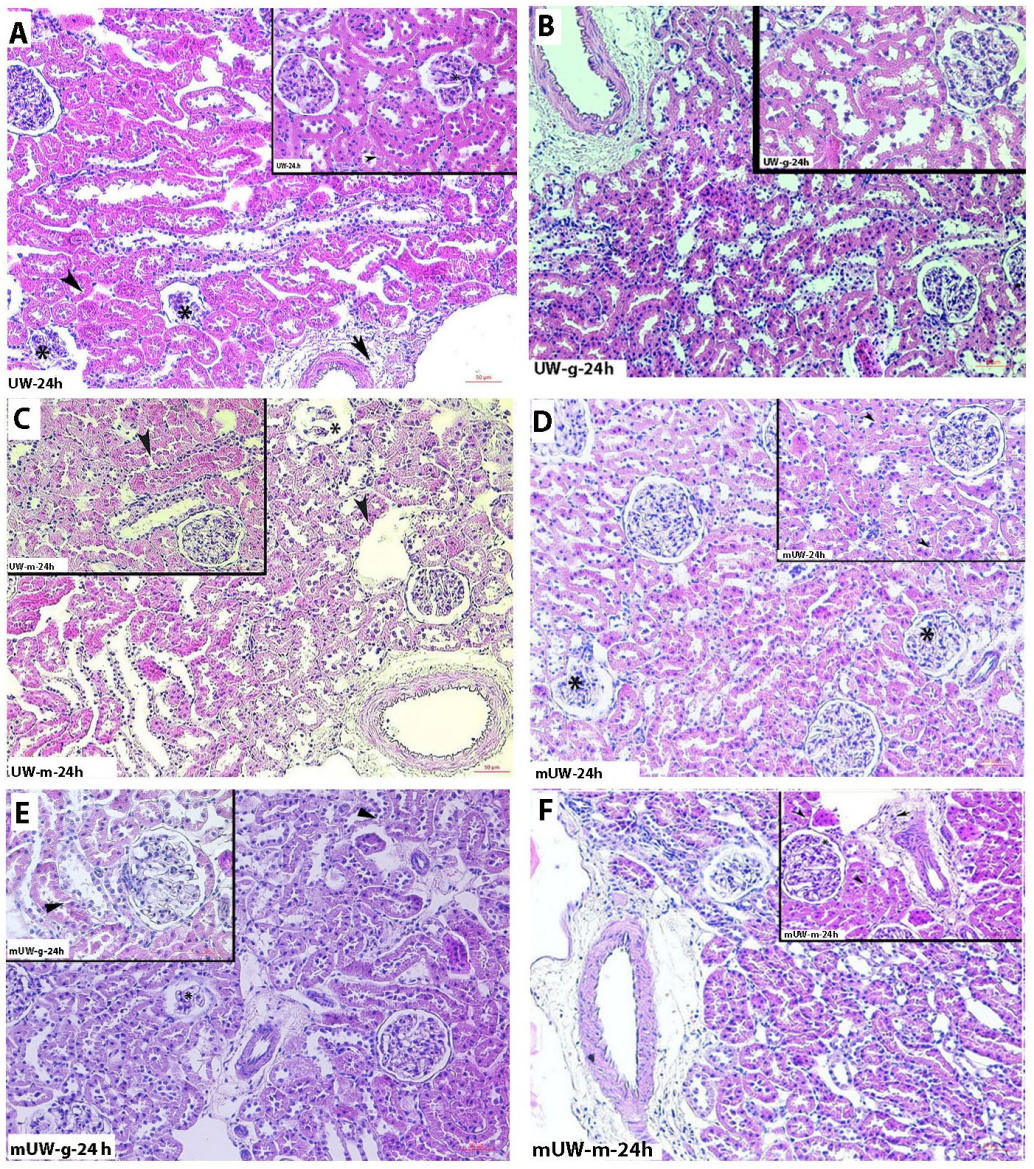
Photomicrographs of kidney tissues stored for 24 hours in experimental kidney preservation solutions. Glomerular degeneration (asterisk), tubular degeneration (arrowhead), inflammatory cell infiltration (short arrow) in UW **(A)** group. Mild degree of tubular degeneration (arrowhead) in UW-g **(B)** and UW-m **(C)** groups. Low degree of glomerular (asterisk) and tubular degeneration (arrowhead) in mUW **(D)** and mUW-g **(E)** groups. Low degree of glomerular degeneration (asterisk), tubular degeneration (arrowhead) and inflammatory cell infiltration (short arrow) in mUW-m Group **(F)**. H&E staining, A,B,C,D,E,F x200 magnification, insets: x400 magnification.

**Fig 4:**
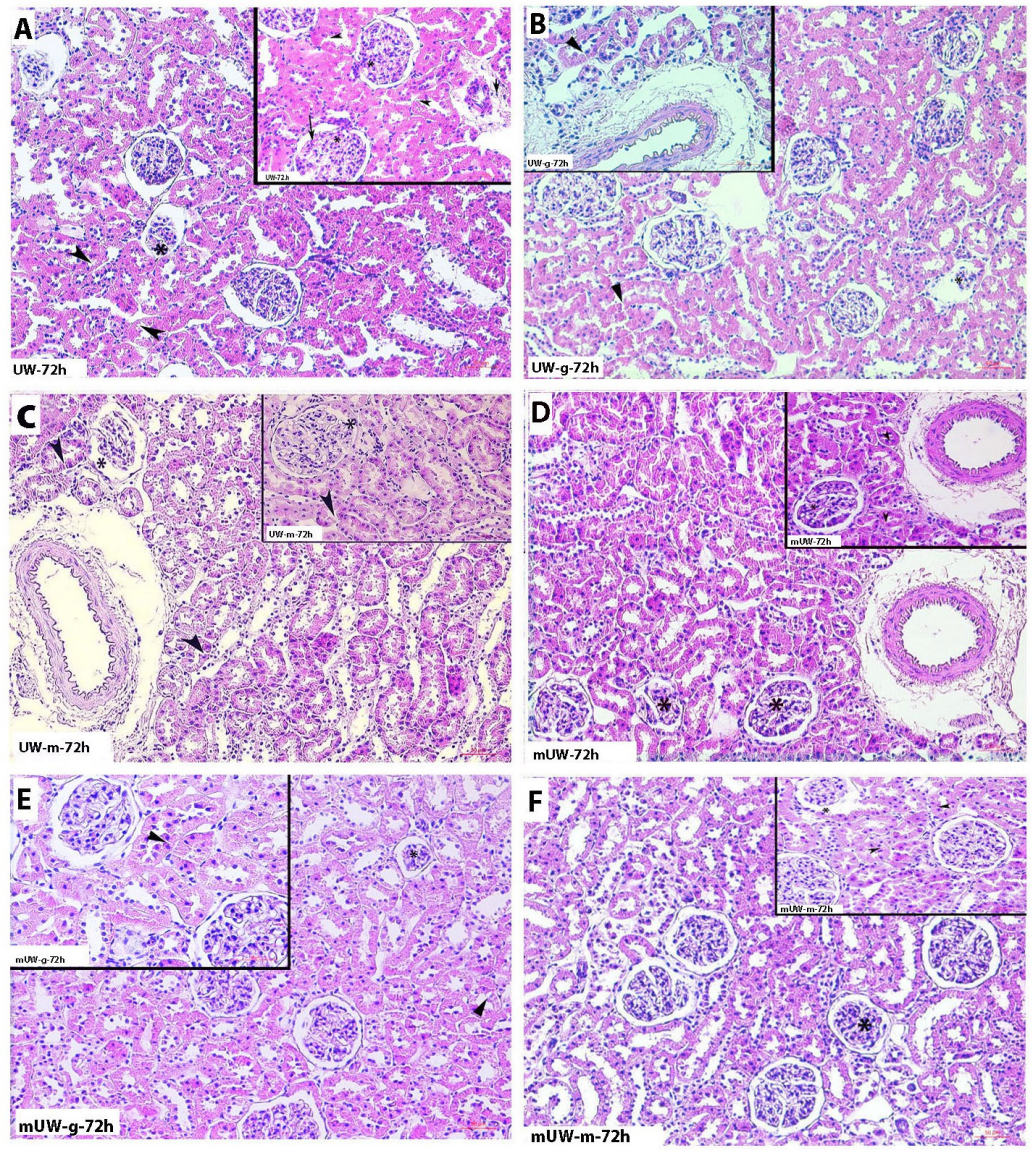
Photomicrographs of kidney tissues stored for 72 hours in experimental kidney preservation solutions. Severe glomerular degeneration (asterisk), tubular degeneration (arrowhead) and inflammatory cell infiltration (short arrow) in UW **(A)** group. Glomerular (asterisk) and tubular degeneration (arrowhead) in UW-g **(B)** and UW-m **(C)** groups. Both the extent and the degree of renal glomerular (asterisk) and tubular degeneration (arrowhead) were apparently lower in mUW **(D)** and mUW-g **(E)** groups. Mild glomerular (asterisk) and tubular degenerations (arrowhead) in mUW-m **(F)** group. H&E staining, A,B,C,D,E,F x200 magnification, insets: x400 magnification.

Histopathologic examinations revealed that tubular and glomerular degenerations, as well as inflammatory cell infiltration, were increased in the UW group with an increasing preservation period (Figs 1–4A). Severe degeneration of renal tissue was noticed at 72 h of the storage period in the UW group (Fig 4A). Vacuolated tubular cells, as well as decreased Periodic acid-Schiff (PAS) positivity of renal tubules and glomerular degeneration, described a prominent tissue injury at 72^nd^ h of this group. Although a mild degree of cellular degeneration in renal tubules was depicting slight damage, in the 2^nd^ h of the UW-m group (Fig 1C), 24^th^ and 72^nd^ h of the UW-m group presented combined moderate damage in the glomerular and renal tubules (Figs 3 and 4C). The mUW solution provided good preservation for all time intervals when compared with UW and UW-m solutions. A mild degree of vasocongestion and inflammatory cell infiltration was seen in all time intervals of the mUW group (Figs 1–4D). Based on light microscopical investigations, preservation in mUW-m solution was much more effective in all time intervals, significantly at 72^nd^ h of preservation (Figs 1–4F).

In the UW-g group, the low tissue injury concerning renal tubules and glomeruli in 2^nd^ h (Fig 1B) tended to increase in the 10^th^, 24^th^, and 72^nd^ h of preservation period (Figs 1–4B). Microscopic investigations on mUW-g group revealed a good tissue preservation compared to UW, UW-g, and UW-m especially in 72^nd^ h (Figs 1–4E).

**Fig 5:**
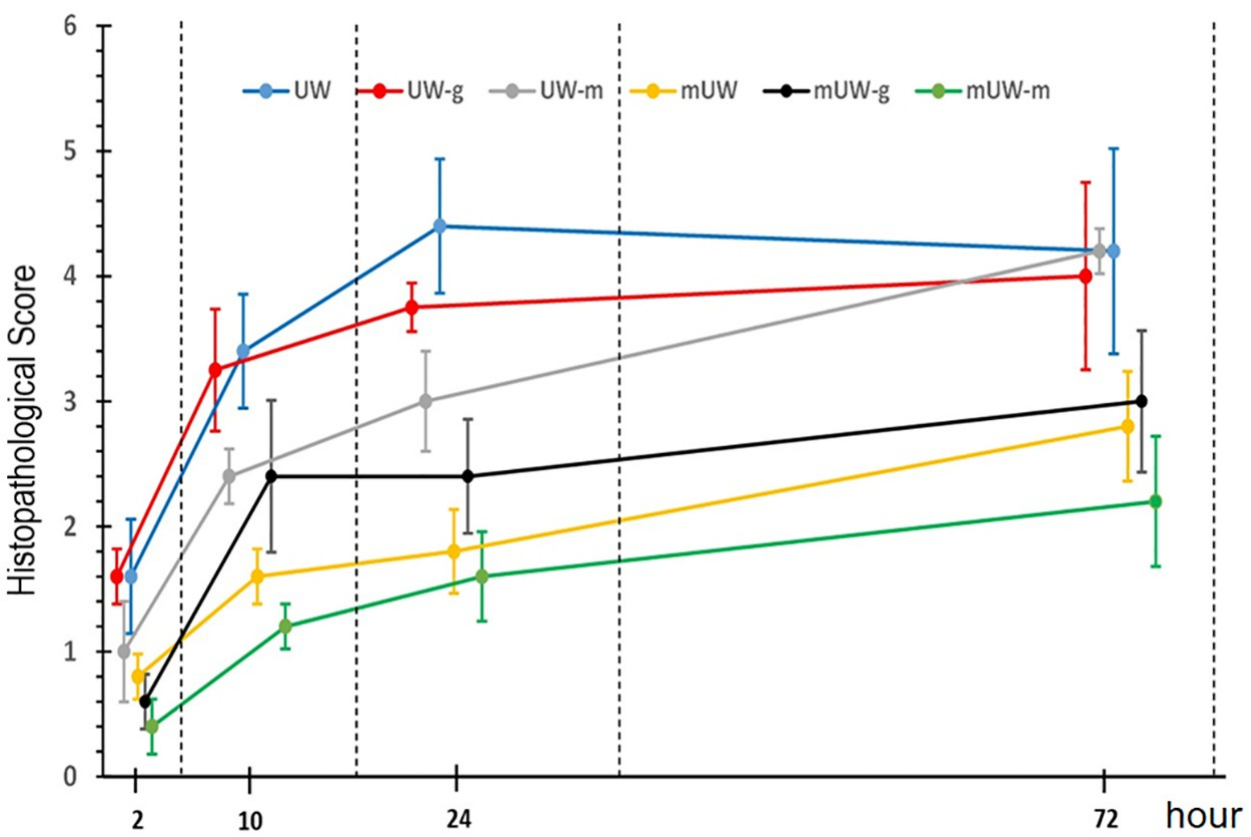
Microscopic score damage of kidney tissue at different time. Data are given as mean±SEM.

At the 2^nd^ h, there was no significant difference in the histopathological score of all experimental groups (Fig 5). At the 10^th^ h of preservation, all groups presented an increase in histopathological score. Tissue damage in mUW, mUW-m and mUW-g groups were comparatively low compared to UW group (Fig 5). Kidney sections from 24 h of preservation groups presented an increased level of tissue damage, more prominently at UW, UW-g and UW-m groups (Fig 5). At 72^nd^ h, the histopathological score was quite increased at UW, UW-g and UW-m groups although score value was decreased in mUW and mUW-m and mUW-g groups (Fig 5).

#### Transmission electron microscopical investigations

**Fig 6:**
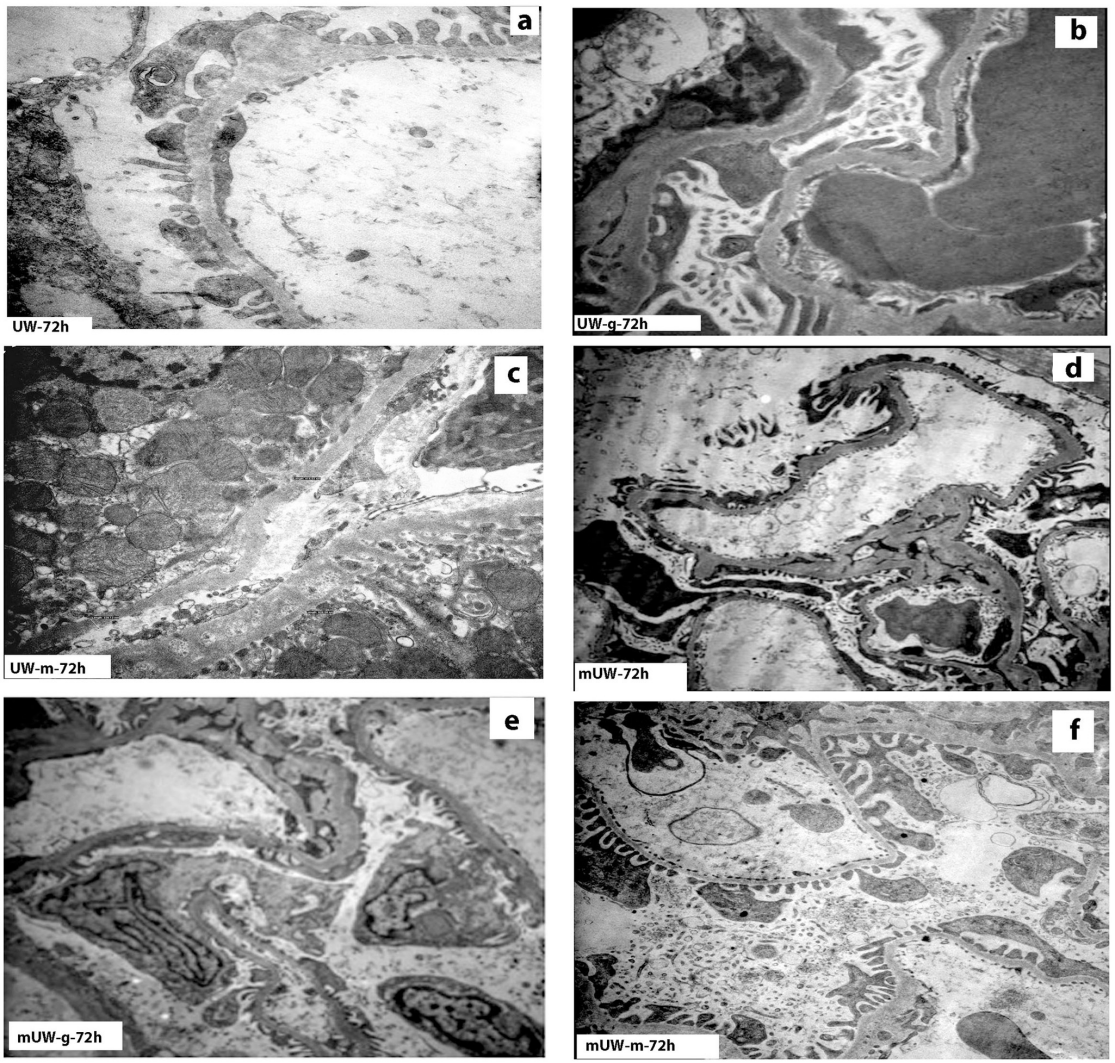
Electronmicrographs of kidney tissue in all experimental solution groups. Kidney tissue damage was ultrastructurally characterized by effacement of pedicels in podocytes, thickening of glomerular basal laminae in UW **(A)** group. Degenerated podocytes and disturbed glomerular basal membranes were observed in UW-g solution group **(B)**. Mildly degenerated podocytes were reflecting low degree of glomerular injury in UW-m group **(C)**. Minimal proximal tubular degenerations were also noticed in this group. Mild degree of podocytes degeneration and moderately thickened glomerular basal laminae in mUW **(D)** and mUW-g **(E)** groups. Mild podocytes degeneration and thickening of glomerular basal laminae in mUW-m **(F)** group. (original magnifications; UW group; x20.000, UW-g group; x20,000, UW-m group; x5000, mUW; x7,500, mUW-g; x7,500, mUW-m; x12,000).

At the 72^nd^ h, electronmicrographs from UW group depicted localized degenerations at podocytes with thickened and humped glomerular basal membranes (Fig 6A). Mesangial proliferation was an important ultrastructural finding indicating the tissue damage in this group. UW-m ultrathin sections presented podocytic degenerations (Fig 6C) with podocytes housing electron-dense residual bodies. Thickened and humped glomerular basal membranes were encountered in the ultrathin sections. Electron micrographs of UW-g group presented damaged podocytes with disturbed glomerular basal membranes (Fig 6B). The mUW group presented giant and double nucleated podocytes with an increase in the mesangial matrix (Fig 6D). Glomerular basal membranes with irregular contours were thickened (Fig 6D). Although podocytic degenerations were still noticed in mUW-g and mUW-m groups, the prevalence of thick and humped glomerular basal membranes was low (Fig 6E and 6F). The tubular structures and mesangial matrix were mostly reflecting normal ultrastructure in mUW-m group (Fig 6F).

**Fig 7:**
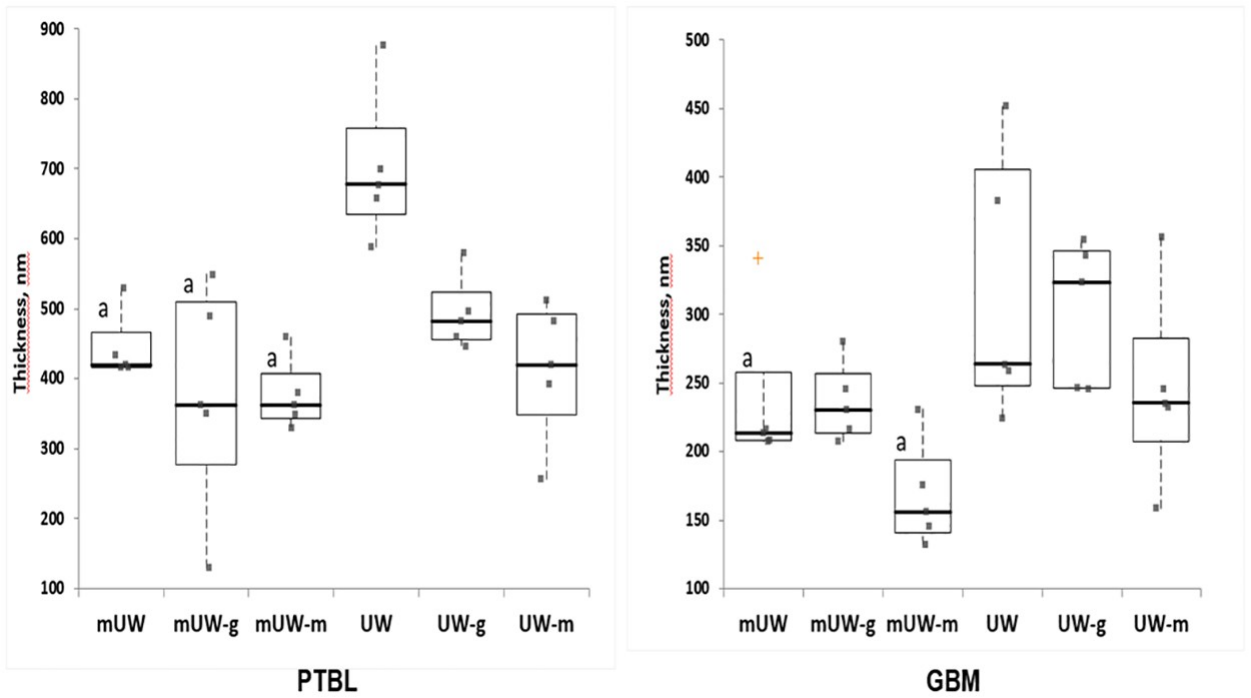
The PTBL and GBM thickness measurement from 72 ^th^ hour of the kidney samples within preservation solutions. a: Significantly different from UW group (p<0.05).

There was no significant difference in the thickness of the glomerular basal membrane of UW, UW-m, and UW-g groups. The thickness of the GBM was decreased in mUW, mUW-m and mUW-g groups (Fig 7). The thickness of PTBL was increased in UW group. The thickening was significantly reduced in UW-g, UW-m, mUW, mUW-m and mUW-g groups compared to UW group (Fig 7).

## Discussion

In this present study for the first time, we changed the content of the standard UW solution and added new agents (melatonin and glucosamine) to preserve kidney tissues for longer than the current preservation period. The protective effect of new preservation solutions on kidney tissues was evaluated in detail using biochemical and microscopical techniques. In animal models the protective effect of preservation solutions were analyzed in different time intervals for up to 72 h [36]. In the present study we observed that the combination of UW and HTK supplemented with melatonin effectively protects kidney tissues for up to 72 h.

Extending the time required for transplantation without significantly harming the organ is of great importance to organize staff and laboratories, transfer organs, and conduct all the required laboratory tests [11]. Therefore, the development of effective preservations solution is the milestone for successful organ transplantation.

We measured LDH activity and protein concentration that was released from the tissue to preservation solution to evaluate renal cell injury. At 2 h of preservation, although proteins released from tissues were measurable but LDH activity was not detectable in all groups. In microscopical evaluation, there were no significant changes suggesting that the tissue maintained its integrity both biochemically and morphologically. However, at 10 h of preservation, biochemical and morphological deterioration can be observed in kidney tissues. This finding suggests that standard UW solution can be limited in effective preservation but mUW and mUW supplemented with melatonin and glucosamine may be more effective since LDH activity was not detected in mUW-m and mUW-g at 10 h of preservation time and tissue damages in mUW-m and mUW groups were comparatively low.

The compositions of preservation solutions vary; however, being hyperosmolar to prevent edema and having a buffering capacity at a favorable pH are common features of different preservation solutions [17,18]. The osmolarity of the modUW solution (358 mOsm) was higher than the standard UW solution (320 mOsm) and a new developed Baskent University Preservation Solution (333 mOsm) [37] but similar to Euro Collins (EC) (355 mOsm) [38] and to Celsior solution [39]. Although UW has been accepted as the ‘gold standard’ solution in solid organ preservation, its high viscosity due to the colloidal structures that were intended to act as a buffer for hydrostatic pressure [40] is accepted as the main disadvantage of the solution [18]. Additionally, it has been shown that UW induces erythrocyte aggregations [41] which might block the microcirculation of solid organs. HTK solution has been successfully used in organ preservation since the 1980s. The main advantage of the HTK solution is the lower risk of biliary complications in liver transplantation [42]. Histidine is a very potent buffer and in comparison to UW, HTK solution has very low viscosity and showed the same efficacy and safety profiles as UW in randomized prospective trials [18]. We hypothesized that decreasing the HES level or changing the colloidal structures might decrease viscosity and facilitate the microcirculation of solid organs. Therefore, for the first time we decreased the concentration of HES by 20% and added histidine to the UW solution. Both light and electron microscopic findings of this study showed that the new UW solution (modified UW) is more effective than the standard UW solution.

Melatonin (N-acetyl-5-methoxytryptamine) is a powerful lipophilic antioxidant molecule and mainly secreted from the pineal gland [43]. It protects subcellular structures effectively by up-regulating heat shock proteins and scavenging free radicals [29,44,45]. The addition of melatonin to the mUW solution protected kidney tissues effectively at 24 and 72 h of preservation. The addition of melatonin and glucosamine to UW and mUW solutions showed a different effect on kidney tissues. We observed that while melatonin protected tissue effectively particularly when combined with the mUW; glucosamine was less effective than melatonin. The ultrastructure of kidney tissues examined by electron microscope showed that the mUW and particularly mUW plus melatonin solution protected kidney tissues effectively up to 72 h. This was evident in all groups containing melatonin particularly combined with modified UW solution. The protective effect of mUW-m solution on kidney tissue was evident particularly on podocytes and GBM. Podocytes are essential for the integrity of the glomerular filtration barrier and its degeneration drives proteinuria [46]. Recently Naik et al [47] showed that early after renal transplantation, accelerated podocyte loss is associated with long-term allograft loss of function. Several studies have shown the association of proteinuria with decreased graft survival and demonstrated that even modest proteinuria is related to graft loss [48–50].

Besides the total protein released from tissue to preservation solutions at different time intervals, we further determined these proteins using proteomic techniques. Among the 29 detected proteins peroxiredoxin-6, fascin, and midkine can be evaluated as potential biomarkers of kidney injury prior to transplantation.

In our previous study, we showed that peroxiredoxin-6 is released from human kidney tissue to preservation solution [26]. Peroxiredoxin-6 is an antioxidant enzyme and its expression is increased with the severity of inflammation. It has a protective role in kidney tissue. For example, Lee et al showed that the overexpression of peroxiredoxin-6 attenuates the lipopolysaccharide-induced acute kidney injury through the inactivation of p38 MAPK (mitogen-activated protein kinase) and JNK (c-Jun amino terminal kinase) pathways [51].

Midkine is a heparin-binding growth factor and has various biological functions particularly in the nervous system, malignancies, and inflammations. In the kidney, midkine is mainly synthesized in proximal tubular cells. The oxidative factors induce the expression of midkine through hypoxia-inducible factor-1α [52]. It has been shown that midkine expression is increased in proximal tubules after kidney ischemic reperfusion injury [53].

### Limitation of the study

In the present study, we used rat kidney tissues to evaluate the protective effects of the modified UW solution combined with melatonin and glucosamine. We are aware that the potential protective effects of these solutions may be different on human kidney tissues. Additionally, we used only male rats and did not re-transplant kidney tissues and therefore the lack of kidney functions data and electrolyte levels are the limitations of the study. However, our results demonstrate valuable information for future studies that will include the transplantation of the preserved kidneys to the rats and assessing the potential protective effects *in vivo*.

## Conclusion

Serious damage occurs after periods of preservation longer than 24 h in UW solution. Based on microscopical investigations, cytoplasmic vacuolization in renal tubular cells, as well as degenerated glomeruli indicated a prominent tissue injury especially at 72nd h of UW group. The protective effect of mUW solution compared to UW solution was remarkable for all time intervals in renal tissue, including 72 h. Based on the histopathological scoring; mUW-m solution was the most potent renal tissue preservation solution in all experimental groups. To conclude, mUW-m solution at low temperature was very effective and suitable solution to protect renal tissues for up to 72 h.

## Supporting information

S1 Data LDH activity, total protein level, histopathological score, GBM and PTBL of kidnye tissue preserved in different preservation solution. (XLSX)

S1 Raw image 2D gel image of protein/peptide profiles deteceted in preservation solution. (TIF)
