# Supplementary figures and images for: Melatonin in preservation solutions prevents ischemic injury in rat kidneys
Source: PLoS One. 2022 Aug 31;17(8):e0273921. doi: 10.1371/journal.pone.0273921 (PMC9432748; doi:10.1371/journal.pone.0273921)

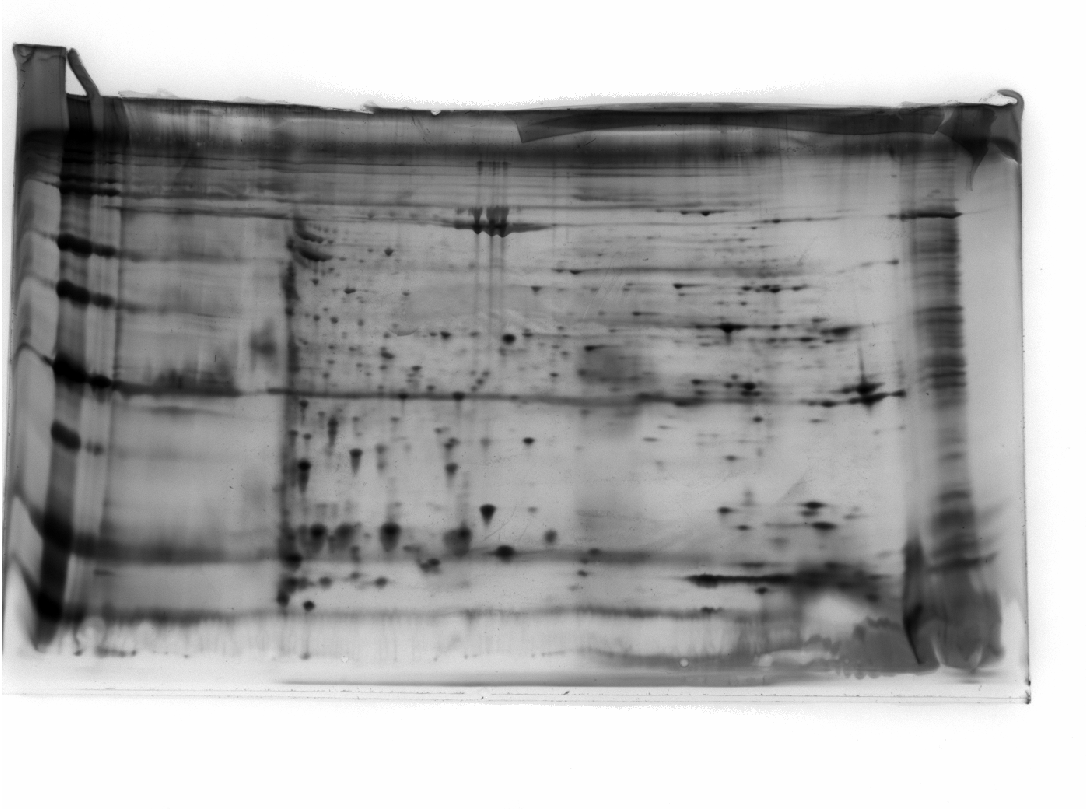

Supplement: S1 Raw image — (TIF) [file pone.0273921.s002.tif]
